# Occupational Hand-Related Injuries at a Major Tertiary Care Burn and Reconstructive Center in Pakistan

**DOI:** 10.7759/cureus.10444

**Published:** 2020-09-14

**Authors:** Dujanah S Bhatti, Nur U Ain, Maryam Fatima

**Affiliations:** 1 Plastic and Reconstructive Surgery, Rawalpindi Medical University, Rawalpindi, PAK; 2 Plastic and Reconstructive Department, Holy Family Hospital, Rawalpindi, PAK; 3 Burn and Reconstructive Department, Holy Family Hospital, Rawalpindi, PAK

**Keywords:** occupation, hand injury, burn, fracture

## Abstract

Introduction

Work-related hand injuries are usually a consequence of mechanical force on hand. This study retrospectively investigated the occurrence of work-related hand injuries in patients belonging to different age groups, gender, educational status, occupation, etc.

Methodology

This cross-sectional study was conducted from October 2018 to December 2019 at the Department of Burn and Plastic Surgery, Holy Family Hospital, Rawalpindi, Pakistan. Demographic and clinical characteristics were gathered through a structured questionnaire based on relevant literature. Patients were assessed using the purposive sampling technique and written informed consent was taken from each participant. A p-value of <0.05 was considered statistically significant. Statistical tests were performed using the Statistical Package for the Social Sciences (SPSS).

Results

One hundred and twenty participants were interviewed, and 87.5% of patients did not have a vascular injury, and 62.5% of patients had an associated fracture. Over half of them (57.5%) had injuries of their dominant hands. Most of the injuries (67.5%) involved fingers while the rest of the injuries were found either in the palm or the dorsum of the hand. Among the causes of accidents, the majority didn't wear or wore unfitted gloves (76.6%) and the main causes of the hand injuries were related to defects in the workplace (53.3%). Lack of concentration (11.7%), wearing loose or unfitted clothes or jewelry (19.2%), lack of machine maintenance (29.2%), and a patient’s chronic disease (1.66%) were among the less frequent causes.

Conclusion

It is important to understand the relationship between occupational and hand injuries. It provides an insight into the lack of protection and guidance of workers.

## Introduction

The hand is the most intricate body part consisting of 20 muscles, 27 bones, numerous tendons, and a network of vessels and nerves. It is the most frequently used body part in our various actions; hence, it is the most susceptible to get injured [1]. One of the leading causes of hand injuries is work-related accidents [2]. According to a study conducted in the US, 35.1% of employees fall victim to work-related injuries [3]. There is a profound burden imposed on the health as well as the socioeconomic status of a person who suffers from such an injury [4]. The loss of productivity that ensues also affects their psychosocial well-being.

An occupational hand injury can vary from simple injuries such as isolated tendon injury to a severely mangled hand [5]. Work-related hand injuries are usually a consequence of mechanical force on hand [6]. The hand can also be subjected to a thermal, chemical, or electrical injury at the workplace. The factors which lead to such injuries include inattention, careless use of machinery/instrument, lack of maintenance of the workplace equipment, lack of personal protective equipment, working overtime, rushing, lack of proper training, etc. [7].

Work-related hand injuries can significantly reduce the quality of life. Studies reveal that with work-related hand injuries, the patient not only loses livelihood, but sometimes it can also cripple the ability to carry out daily routine tasks [8]. It was also noted in a study hand injuries create delays in returns to work, adding the financial burden on the patients and their families [9-11].

This study retrospectively investigated the occurrence of work-related hand injuries in patients belonging to different age groups, gender, educational status, occupation, etc. This study also explored various causes that lead to work-related hand injuries and a number of variables that influence such injuries.

## Materials and methods

This cross-sectional study was conducted from October 2018 to December 2019 at the Department of Burn and Plastic Surgery, Holy Family Hospital, Rawalpindi, Pakistan. The study was approved by the Ethics Review Committee of Rawalpindi Medical University.

The authors developed a structured questionnaire (see appendix) based on relevant literature [12, 13]. The instrument was further pre-tested on 20 patients prior to data collection to assess presentation, acceptability, and ease of understanding of the questions. The questionnaire required little modification prior to use. Patients were assessed using the purposive sampling technique and written informed consent was taken from each participant. Patients were also informed of the study's purpose.

Patients were included in this study only if they were (i) injured at work; (ii) presented in the emergency department; (iii) scheduled for a follow-up visit after one and two weeks from the date of injury; and (iv) had injury limited to hand (including fingers) and wrist zone. The patients under the age of 18 years were only included if they were accompanied by their parents/guardians and were interviewed in the presence of their attendants. However, patients or attendants (in case of a minor patient) who could not provide informed consent were excluded from the study.

Information was collected on the following three sections:

i) Demographic information and other factors: patients were asked to describe their age, gender, education, occupation type, means of transport, and smoking status. 

ii) Clinical characteristics: patients were either asked or confirmed through their medical history regarding the type of injury (vascular, nerve), fracture on X-ray, event by exposure, the affected hand is either dominant or non-dominant, type of injury, injuries involved hand, digits involved in hand injuries, time of presentation and place of first aid.

iii) Patients were asked to confirm what are the causes of occupational injuries.

Descriptive statistics were calculated using frequencies and percentages. A Chi-square test was used to assess the association between socio-demographics and clinical characteristics of patients. A p-value of <0.05 was considered statistically significant. Statistical tests were performed using the Statistical Package for Social Sciences (SPSS version 26.0; IBM Inc., Armonk, US).

## Results

A total of 120 participants were interviewed. About half (47.5%) of the participants were less than 18 years of age. About 25% of patients fell in the age group ranging from 18 years to 35 years, and 15% belonged to the age group ranging from 35 years to 50 years old. Only 12.5% were 50 years of age or above. Most of the study participants were male (82.5%). Over half of the participants were non-skilled workers (52.5%) and were illiterate (62.5%). Most of the study participants (52.5%) were smokers; more than half (30%) of them smoked more than 20 packs per day (Table 1).

**Table 1:** Sociodemographics and smoking status of patients (n=120)

Description		Frequency	Percentages
Age	14 years - 18 years	57	47.5
	18 years - 35 years	30	25.0
	35 years - 50 years	18	15.0
	>50 years	15	12.5
Gender	Male	99	82.5
	Female	21	17.5
Patient education	Illiterate	75	62.5
	Literate	45	37.5
Patient occupation	Professional	27	22.5
	Skilled	30	25.0
	Non-skilled	63	52.5
Means of transport	Private car	21	17.5
	Taxi/cab	36	30.0
	On foot	51	42.5
	Ambulance	12	10.0
Smoking status	Non-smoker	57	47.5
	Smoker >20 per day	36	30.0
	Smoker <20 per day	27	22.5

We compared patients aged below 50 with those above 50 as the former group consists of patients that are most active and lack comorbidities while the latter group comprises individuals that are less active and might be suffering from certain comorbidities. It was observed that patients aged 50 and below suffered an injury in their dominant hand, had isolated hand injury, and the cause was mostly related to the use of a machine or a tool. They also had evidence of fractures on X-rays. Other causes for males in addition to the use of machinery included interpersonal violence etc. For females and illiterate patients, an isolated, non-dominant hand was found to be involved in injury with evidence of fracture on X-rays, mostly due to the use of machines or tools. Vascular and nerve injury, if present, was more prevalent in female patients. Literate patients suffered from an isolated hand injury in the dominant hand, and causes were other than the use of machines or tools. Skilled and semi-skilled workers tended to have isolated injuries in the dominant hand, while unskilled workers most affected hand was non-dominant. Moreover, unskilled laborers tended to suffer from vascular injury and had radiological evidence of fracture (Table 2).

**Table 2:** Association between socio-demographics and clinical characteristics of patients

Demographic characteristics	N	No n (%)	Yes n (%)	p-value	No n (%)	Yes n (%)	p-value	No n (%)	Yes n (%)	p-value
		Vascular injury			Nerve injury			Fracture on X-ray		
Age										
50 and below	105	90 (75.0)	15 (12.5)	0.118	87 (72.5)	18 (15.0)	0.074	30 (25.0)	75 (62.5)	0.001
Above 50	15	15 (12.5)	0 (0.0)		15 (12.5)	0 (0.0)		15 (12.5)	0 (0.0)	
Gender										
Male	99	99 (82.5)	0 (0.0)	0.001	99 (82.5)	0 (0.0)	0.001	21 (17.5)	54 (45.0)	0.001
Female	21	6 (5.00)	15 (12.5)		3 (2.5)	18 (15.0)		0 (0.0)	45 (37.5)	
Education										
Illiterate	75	60 (50.0)	15 (12.5)	0.001	57 (47.5)	18 (15.0)	0.001	0 (0.0)	75 (62.5)	0.001
Literate	45	45 (37.5)	0 (0.0)		45 (37.5)	0 (0.0)		45 (37.5)	0 (0.0)	
Patient occupation										
Skilled/semi-skilled	57	57 (47.5)	0 (0.0)	0.001	57 (47.5)	0 (0.0)	0.001	45 (37.5)	12 (10.0)	0.001
Unskilled labor	63	48 (40.0)	15 (12.5)		45 (37.5)	18 (15.0)		0 (0.0)	63 (52.5)	
Demographic characteristics	N	No n (%)	Yes n (%)	p-value	By use of machine/ tool n (%)	Other accidental/ interpersonal violence n (%)	p-value	Isolated hand n (%)	Associated injury n (%)	p-value
		Affected hand is dominant			Event by exposure			Type of injury		
Age										
50 and below	105	36 (30.0)	69 (57.5)	0.001	66 (55.0)	39 (32.5)	0.001	105 (87.5)	0 (0.0)	0.001
Above 50	15	15 (12.5)	0 (0.0)		0 (0.0)	15 (12.5)		6 (5.0)	9 (7.5)	
Gender										
Male	99	51 (42.50)	48 (40.0)	0.001	45 (37.5)	54 (45.0)	0.001*	90 (75.0)	9 (7.5)	0.166
Female	21	0 (0.0)	21 (17.5)		21 (17.5)	0 (0.00)		21 (17.5)	0 (0.0)	
Education										
Illiterate	75	6 (5.0)	69 (57.5)	0.001	66 (55.0)	9 (7.5)	0.001	75 (62.5)	0 (0.0)	0.001
Literate	45	45 (37.5)	0 (0.0)		0 (0.0)	45 (37.5)		36 (30.0)	9 (7.5)	
Patient occupation										
Skilled/semi-skilled	57	51 (42.5)	6 (5.0)	0.001	3 (2.5)	54 (45.0)	0.001	48 (40.0)	9 (7.5)	0.01
Unskilled labor	63	0 (0.0)	63 (52.5)		63 (52.5)	0 (0.0)		63 (52.5)	0 (0.0)	

**Figure 1:**
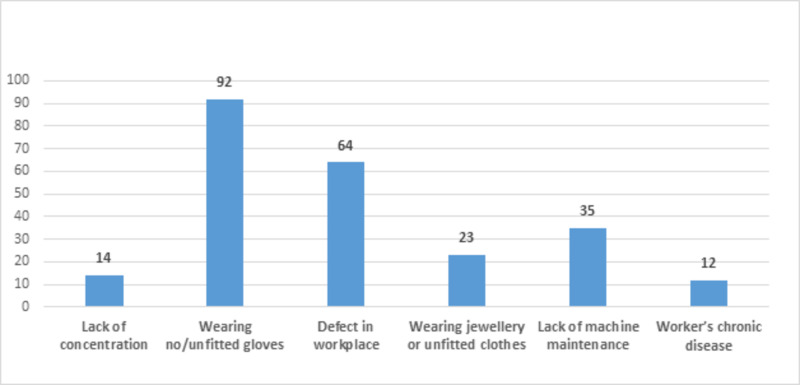
Cause of occupational injury (n=120)

Among the causes of accidents, the majority mentioned that not wearing or wearing unfitted gloves (76.6%) and defects in the workplace (53.33%) were the main causes of hand injuries. Lack of concentration (11.66%), wearing loose or unfitted clothes or jewelry (19.16%), lack of machine maintenance (29.16%), and a patient’s chronic disease (1.66%) were among the less frequent causes (Figure 1).

Over half of the study subjects (57.5%) were injured in their dominant hands. Fifty-one percent of patients presented to the emergency room on foot, followed by taxi and private cars. Over half of the participants (55%) were using machinery or tools during the injury. Patients facing injury due to accidents such as falls were 33.5 %, while 12.5% of injuries were attributed to interpersonal violence. About 65% of the study population presented from 2 pm to 10 pm. A minority (7.5%) presented overnight. Less than half of the participants (35%) received first aid measures after the injury, and more than half of them (63.3%) reached the hospital by taxi (Table 3).

**Table 3:** Clinical information of patients (n=120)

Description		Frequency	Percentages
Vascular injury	Yes	15	12.5
	No	105	87.5
Nerve injury	Yes	18	15.0
	No	102	85.0
Fracture on X-ray	Yes	75	62.5
	No	45	37.5
Event by exposure	By use of machine/tool	66	55.0
	Other accidental (e.g., fall)	39	32.5
	Interpersonal violence	15	12.5
Affected hand	Dominant	69	57.5
	Non-dominant	51	42.5
Type of injury	Isolated hand injury	111	92.5
	Associated injury	9	7.5
Injuries involving hand	Finger	81	67.5
	Palm	24	20
	Dorsum	27	22.5
Digits involved in hand injuries	Little	12.0	10.0
	Ring	42.0	35.0
	Middle	36.0	30.0
	Index	54.0	45.0
	Thumb	33.0	27.5
Time of presentation	8 am - 2 pm	33	27.5
	2 pm - 10 pm	78	65.0
	10 pm - 8 am	9	7.5
Place of first aid	At the workplace	42	35.0
	Outside workplace	78	65.0

Most of the injuries (67.5%) involved fingers, while the rest of the injuries were found either in the palm or the dorsum of the hand. The little finger was least commonly (10%) involved in the injuries, while the index finger was most commonly involved (45%). The incidence of the involvement of the rest of the digits was almost equal, i.e., ring finger (35%), middle finger (30%), and thumb (27.5%). In 62.5% of workplace hand injuries, there was radiographic evidence of fracture. Most of the patients did not have any vascular (87.5%) or nerve (85%) injury.

## Discussion

The patient presenting with hand injuries can serve as clinical data on occupation incurred trauma and a surveillance tool to identify the likelihood of job categories and the related incidence of injuries. It also elucidates the most noticeable category of injury, along with its associated fractures and neurovascular damage. In our study, the majority (47.5%) of the patients presenting in our outpatient clinic or the emergency department were under the age of 18 years. The elderly population had an exceedingly small fraction to present in our study. These figures were consistent with a study done by Smith et al. in which the largest pool of patients was the younger age group (less than 18 years of age) [14]. This could be because, in our region, child-driven labor is widely practiced, despite restrictions [15]. The younger population also lacks the proper skills and training to handle equipment that is associated with a higher risk of occupational injuries. Warner et al. showed similar results - younger patients were prevalent [16]. The older age group, i.e., 50 years and more, have decreased ability to take part in occupational activities, and most of them retire by the age of 60 years; hence this age group showed less incidence of occupation-related hand injuries [17]. Gender distribution has a higher ratio of male to female in our sample. This is similar to other studies where males were given more manual-intense labor [18]; this could attribute to the greater male population in our study. We observed a gender variance with the male predominance of occupation-related hand injuries, which corroborates with the study by Farhad et al. [19]. The dominance of the male gender can be due to the cultural and social norms of our country where the male is the bread earner of the family, and financial aspects of the household are the responsibility of the male. Therefore, males have a much higher employment rate than women and thus are more prone to getting occupation-related hand injuries.

Concerning the literacy status of our participants, Illiterate and non-skilled individuals made most of our patients. This was consistent with a study done by Shankar et al. in which a significant proportion of injured patients were of the same category (41.3% illiterate and 41.5% non-skilled) [20]. This suggested that the educational status of individuals has a direct correlation with occupation-related hand injuries. Illiterate and non-skilled workers were not familiar with safe practices, which makes them susceptible to work-related hand injuries. Our study involved occupations that involved labor-intense manual work that included handy work and operation of heavy machinery. These occupations are vulnerable to injuries such as lacerations, de-gloving injury, fractures, neurovascular compromise, amputation, etc. Singh et al. showed similar results in India [21].

Means of transportation are noteworthy because if the patient reached the hospital by ambulance, one must have received first aid. Contrary to this, there is a time delay which suspends the receiving of the first aid if the patient has used personal transport. Undue delays can be encountered if one takes a cab; however, if the patient uses personal transportation, reaching the hospital happens without such delays. 

Occupational injuries without major vascular and nerve injuries were common in our study. Ninety-two percent of the injured in the studied population had isolated hand injuries. Injuries to nerves only accounted for 18% of the studied population. Similarly, vascular damage was seen in 15%. Lack of proper training in assessment of cutaneous nerve distribution and vascular assessment at the time of initial patient assessment could attribute to missed nerve and vascular injuries. This could be the reason why our study detected only 15% documented nerve damages on presentation. Similar aspects of nerve involvement were seen by a study done by Strong et al. in which definitive diagnostic nerve conduction study had to be employed to find out the exact quantification of the true incidence of peripheral neuropathy. The most frequent non-cutaneous injury documented in our study were bone fractures, accounting for 62%. This could be attributed to the early recognition of fracture on an X-ray or clinical examination. Fracture is more evident in the primary survey and hence rarely missed [22].

The type and intensity of occupation play a major role in occupation-related hand injuries and can be deadly at times. We observed that machine work and the use of hand tools resulted in a little more than half of the work-related hand injuries. These figures are similar to Hunt et al. findings, where they compared fatal and non-fatal workplace injures [23]. Hand lateralization was seen in 69% in our results, like Arifi et al., where involvement of the dominant hand was almost 50%. The resulting negative impact on the vocational and economic status of the patient can lead to anxiety and distress [24]. Sixty-five percent of the patients presented to the medical institution during the afternoon. This could be because the working hours in our region and late presentation could be due to lack of transport availability as most of our patients utilized private cars or preferred walking to the hospital due to lack of any other source of conveyance (42%).

It is estimated that fingers are mostly affected by accounting for 67% of total injuries. Injuries of the hand could be predominant in our population because of male dominance and accidents attained during work involving hand-work. This is supported by Abu-Sittah el al., who showed that injuries to fingers prevailing palm injuries [25]. We also saw that index and ring fingers were commonly affected. These injuries should be immediately taken care of as they can result in grave consequences impacting the functional status of the patient and hence economic and psychosocial well-being. 

It is observed that most of the laborers are smokers, and their engagement in smoking activities while working can lead to distraction and ultimately result in work-related injuries. This is aggravated by the lack of usage of personal protective equipment such as gloves etc. Not wearing gloves or wearing unfitted gloves was found to be the primary cause of occupation-related hand injuries, which contradicts the results of the study done by Jin et al. [26]. Defects in the workplace and lack of machine maintenance were other common causes being supported by findings of other studies [27] but contradict the study conducted by Mostafa et al., where the lack of concentration was found to be a less common cause of work-related hand injuries [28].

## Conclusions

It is important to understand the relationship between occupational activities and hand injuries. It provides an insight into the lack of protection and guidance for workers involved in professions that apply hand-work. It also provides us with a better understanding of the management of such injuries. Documented risk factors can be considered to prevent such occupational injuries in the future.

## Figures and Tables

**Figure 2:**
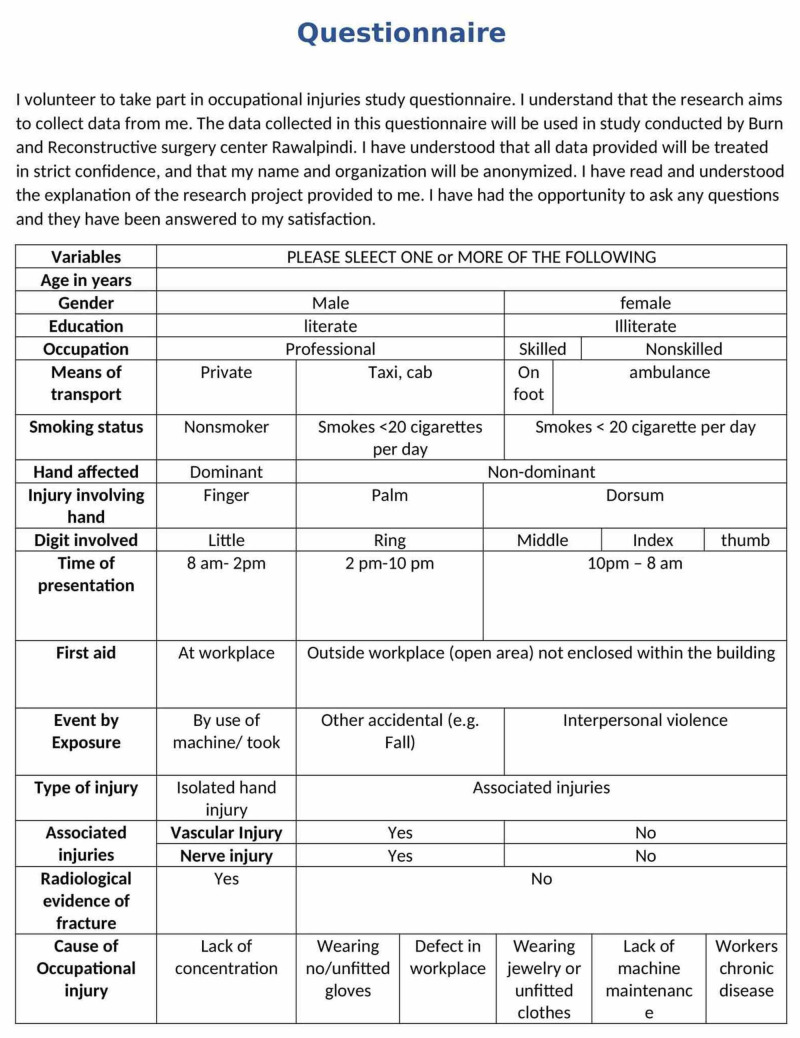
Questionnaire
